# Evaluation of the endoscopic third ventriculostomy success score for stereotactic prepontine stenting in patients with aqueductal stenosis

**DOI:** 10.1016/j.bas.2026.106031

**Published:** 2026-04-05

**Authors:** Moritz Ueberschaer, Katja Wirthensohn, Sebastian Niedermeyer, Robert Forbrig, Niklas Thon, Sabrina Viktoria Kirchleitner, Mathias Kunz, Wolfgang Hitzl, Michael Schmutzer-Sondergeld

**Affiliations:** a Department of Neurosurgery, LMU University Hospital, LMU Munich, Munich, Germany; b Department of Neurosurgery, University Hospital Salzburg, Paracelsus Medical University, Salzburg, Austria; c Institute of Diagnostic and Interventional Neuroradiology, LMU University Hospital, LMU Munich, Munich, Germany; d Department of Neurosurgery, University Medical Center Knappschaftskrankenhaus, Bochum, Germany; e Department of Neurosurgery, University Hospital Marburg, Phillips University Marburg, Germany; f Research and Innovation Management, Biostatistics, Department of Ophthalmology and Optometry, Research Program Experimental Ophthalmology and Glaucoma Research, Paracelsus Medical University, Salzburg, Austria

**Keywords:** ETVSS, Hydrocephalus, Aqueductal stenosis, ETV, Stereotaxy, Endoscopy

## Abstract

**Introduction:**

Stereotactic prepontine stenting (STS) has been shown to be a safe and effective alternative treatment to standard endoscopic third ventriculostomy (ETV) for the treatment of triventricular hydrocephalus (TVH). For ETV a success score (ETVSS) allows risk stratification for treatment failure, which was validated in young patients. For patients with TVH treated with STS a comparable success score was not performed to date. This was the reason why we wanted to test whether the ETVSS is also applicable to this patient cohort.

**Research question:**

Is the ETVSS also eligible for patients undergoing STS?

**Methods:**

TVH patients undergoing either ETV or STS between 2013 and 2024 were included retrospectively. Treatment failure was defined as absence of symptomatic and/or imaging improvement. ETVSS and its predicitive power were calculated for each group and correlated with outcome. Further statistical models were applied to create alternative scores.

**Results:**

50 STS patients had a mean ETVSS of 88.2 ± 3.9% compared to 81.8 ± 16.7% in 97 patients undergoing ETV (p = 0.009). Successful treatment was achieved in 87% of ETV and 96% of STS patients (p = 0.09). Mean ETVSS after successful treatment was 89.1 ± 2.9% versus 81.7 ± 4.1% with treatment failure in the STS and 84.1 ± 13.5% versus 71.7 ± 24.8% in the ETV group (p = 0.02 and p = 0.3). ROC analysis showed varied performance of ETVSS in the STS (AUC = 0.553) and ETV cohort (AUC = 0.766). Univariate analysis showed significant influence of the clivus-basilar artery diameter on treatment success in the ETV group. Identified risk factors did not allow the establishment of new scores.

**Conclusion:**

ETVSS did not enable prediction of treatment success by STS. Individual decision-making is essential, especially in patients with low ETVSS. Future studies must include a more heterogeneous patient population to enable new scoring systems specifically for patients undergoing STS.

## Highlights

• Alternative surgical treatment option for aqueductal stenosis is the stereotactic prepontine stenting (STS) compared to classic endoscopic third venriculostomy (ETV)
• Successful treatment of hydrocephalus was achieved in 87% of ETV and 96% of STS patients
• ROC analysis showed poor performance of ETVSS in the STS and good performance in the ETV cohort
• Univariate analysis and the importance plot showed significant influence of the clivus-basilar artery diameter on treatment success in the ETV cohort

## 1 Introduction

Aqueductal stenosis (AS) is a common pathology leading to hydrocephalus (Cinalli et al., 2011). The treatment of choice in symptomatic patients is endoscopic third ventriculostomy (ETV), which has been shown to be safe and effective (Jiang et al., 2018; Bergsneider et al., 2008). In a recent publication, our group compared outcomes and complications in a cohort of patients who underwent either ETV or stereotactic prepontine stenting as an alternative treatment in patients with triventricular hydrocephalus (TVH) that may reduce the risk of stoma occlusion. We found that both treatment modalities had comparable clinical outcomes and complication rates with slightly lower rates of stoma occlusion after STS not reaching statistical significance (Ueberschaer et al., 2025).

**Fig. 1:**
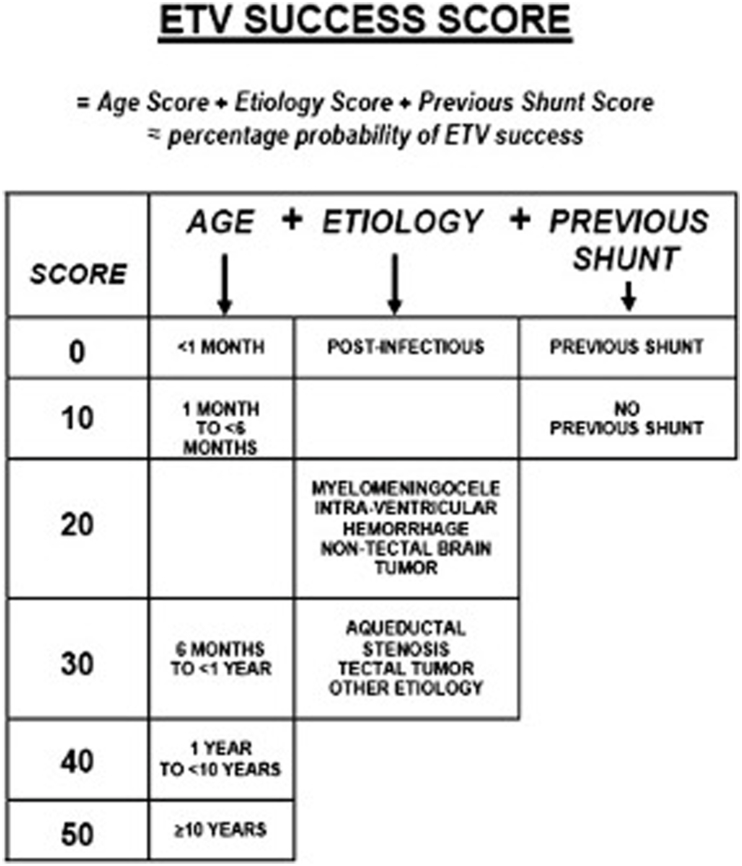
ETV success score, published by Kulkarni et al., in 2009 (Kulkarni et al., 2009). The higher the score, the higher the probability of successful ETV.

The choice of treatment method therefore remains a challenge. A success score for ETV (ETVSS) has previously been developed to predict the risk of treatment failure (Kulkarni et al., 2009) (Fig. 1). Age, etiology of the aqueductal stenosis and a previous shunt contribute to the probability of a successful ETV. While this scoring system has been validated several times in different cohorts (Labidi et al., 2015; Kulkarni et al., 2010; Krause et al., 2024; Adil et al., 2025), we wondered whether it is also applicable to STS. Therefore, we sought to evaluate the score in our own cohort of patients with aqueductal stenosis who underwent STS and compare its performance with that of patients who underwent ETV. The aim of this study was to evaluate the predictive value of ETVSS for STS in patients with aqueductal stenosis and TVH in order to facilitate the decision regarding the treatment modality.

## 2 Methods

We performed a retrospective single center study including all consecutive patients who underwent an ETV or STS procedure due to symptomatic AS between January 2013 and July 2024. Patients had either aqueductal stenosis caused by an arachnoid web or aqueductal stenosis caused by compression of a tumor or a cystic lesion. In order to be able to assess individual ETV success scores, patient age, reason for aqueductal stenosis and previous shunt were documented and scores were calculated as previously described (Kulkarni et al., 2009). The primary outcome (treatment success) was defined as no revision surgery being performed within 6 months due to inadequate treatment of the hydrocephalus. Inadequate treatment was considered as persisting or worsened symptoms and/or absence of flow void signal on MRI after ETV. The local ethics committee approved the study (reference number 22-0511). Surgical treatment decision was made according to the preferences of the surgeon and the patient. As already published by our group either ETV or STS was performed (Ueberschaer et al., 2025). For ETV either the Lotta or the little Lotta endoscopes (Karl Storz SE & Co. KG) were used. The Minop ® endoscope (B.Braun SE) was used for the last patients in series (2023-2024), as the little Lotta was no longer available. After puncturing the ventricle, the endoscope was advanced into the lateral ventricle and further through the foramen of Monro into the third ventricle. Once the important landmarks were visualized, the third ventricular floor was bluntly perforated, followed by dilatation of the stoma with a Fogarty balloon catheter. The prepontine cistern was then inspected to rule out persistent arachnoid webs. If pulsation of the cerebrospinal fluid through the stoma was observed and a free connection to the prepontine cistern was evident, the endoscope was removed. The burr hole was closed using Spongostan or a borehole plate.

**Fig. 2:**
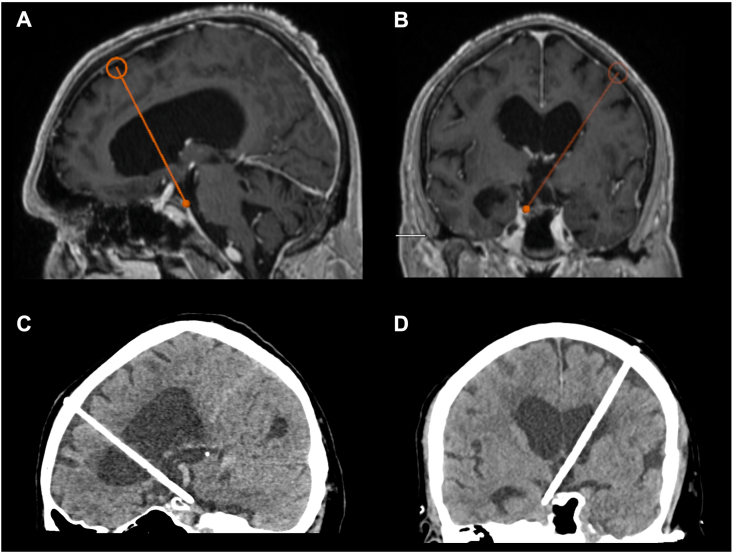
T1-weighted with contrast cMRI scans of the preoperative trajectory planning for an STS in a patient with aqueductal stenosis due to an arachnoid web vie a left frontal trajectory (sagittal and coronal, A and B). Postoperative cCT with the internal shunt catheter tip in the prepontine cistern (sagittal and coronal, C and D).

For minimal-invasive treatment, an internal shunt catheter was implanted stereotactically (STS) to connect the lateral ventricles with the basal cisterns (Schmutzer-Sondergeld et al., 2024a, 2025; Niedermeyer et al., 2024). For this, surgical planning (iPlan stereotaxy; Brainlab, Munich, Germany) was based on a stereotactically localized contrast-enhanced computed tomography (CT) scan (0.6 mm slice thickness) and the preoperative MRI data (T1-weighted with and without contrast, T2-weighted/CISS sequences, contrast-enhanced magnetic resonance angiography), which were co-localized with the CT scan. A 1.3 mm diameter catheter (Becker EDMS ventricular catheter; Medtronic Inc, Dublin, Ireland) was stereotactically implanted via a 2 mm burr hole. Additional catheter perforations were added manually to achieve optimal up- and downstream drainage. The catheter was fixed extracranially with a hemoclip (Titanium Ligation-Clip, 150 mm length, B Braun, Melsungen, Germany) placed orthogonally on the catheter on the calvaria preventing the catheter from sliding into the brain. Above this, a sponge sealant patch (TachoSil®, Takeda Pharmaceuticals, Konstanz, Germany) was attached for adequate closure and additional fixation. Fig. 2 shows the preoperative trajectory planning for an STS in a patient with aqueductal stenosis due to an arachnoid web via a left frontal trajectory (A and B) and the postoperative cCT with the internal shunt catheter (C and D).

### 2.1 Symptom assessment

Headache, gait disturbance, and urinary incontinence were documented from neurological examinations and clinical records at baseline and postoperatively. Cognitive impairment was evaluated using age-appropriate neuropsychological or clinical assessments, and papilledema was assessed by ophthalmological fundoscopy at both time points.

### 2.2 Calculation of evans index

Preoperative cMRI (1.5- or 3.0-T scanners: Magnetom Symphony, Siemens, Erlangen; Signa HDxt; GE Healthcare, Little Chalfont, United Kingdom) routinely included axial T2-weighted sequences (with slice thickness of 2 mm), 3-dimensional T1-weighted sequences before and after intravenous administration of gadopentetate dimeglumine (0.1 mmol/kg body weight; Magnevist; Schering Corporation, Kenilworth, NJ), as well as constructive interference in steady-state (CISS) sequences (with slice thickness of 1 mm). The Evans index (EI) was determined as a marker of pre- and postoperative hydrocephalus on the basis of the performed cMRI images. The EI is calculated as the ratio between the maximum width of the lateral ventricular anterior horns and the maximum inner diameter of the skull on the same slice level with values > 0.3 representing an enlarged ventricular system (Neikter et al., 2020; Toma et al., 2011).

### 2.3 Statistical analysis

The ETVSS was calculated for the ETV and STS groups and compared using Student's *t*-test. A calibration plot was used to estimate the predictive power. For risk factor analyses uni- and multivariate tests were conducted. For correlation analyses Pearson's coefficient *r* was determined. In addition, a receiver operator characteristics (ROC) analysis was performed and the area under the curve (AUC) was calculated to compare the performance of the score for both treatment modalities with GraphPad PRISM 8.0 software (GraphPad, San Diego, CA, USA). Statistical significance was set at p < 0.05. In an effort to create an alternative score, uni- and multivariable logistic regression models with odds ratios and 95% CI were computed. Additionally, gradient boosted trees, decision trees, support vector machines, nearest neighbours classifiers, random forest models, Bayes classifiers and multilayer perceptron neural networks were applied. 10-fold cross-validation was used for model training using 10% of training data as validation set each. A ‘reject option’ was applied, i.e., instead of one cut-off, two cut-offs were applied to the a posteriori probabilities, allowing the models to reject to make a prediction. Statistical significance for all reported tests was determined using two-sided tests and the significance level was set to 5%. Analyses were conducted using STATISTICA 13 (Hill, T. & Lewicki, P. Statistics: Methods and Applications. StatSoft, Tulsa, OK) and MATHEMATICA 13.0 (Wolfram Research, Inc., Mathematica, Version 13.0, Champaign, IL, USA, 2021)

## 3 Results

In total, 147 patients underwent surgery for aqueductal stenosis (AS). In 60/147 patients (40.8%) stenosis was caused by tumor formations (33 gliomas, 18 pineal tumors and 9 metastases). 50/147 (34.0%) patients were treated by STS and 97/147 (66.0%) patients by ETV, respectively. Detailed patient characteristics according to treatment modality are listed in Table 1. The main symptoms of headache, cognitive impairment, gait disorder, urinary incontinence and papilledema have improved remarkably well both in the ETV and STS group (Table 2). In the overall cohort, correlation analyses displayed a significant association of an older patient age (r = 0.3, p = 0.01), an enlarged clivus to basilar artery distance (r = 0.3, p = 0.002) and a decreased postoperative EI (r = 0.2, p = 0.04) with higher ETVSS scores suggesting successful hydrocephalus treatment.

**Table 1:** Patient characteristics according to treatment modality.

	STS group	ETV group	*p value*
**n total (%)**	50 (34)	97 (66)	
**Sex, n (%)**			
Male	26 (52)	51 (53)	0.99
Female	24 (48)	46 (47)	
**Reason for AS, n (%)**			
Solid tumor	33 (66)	20 (21)	**<0.0001**
Cystic tumor	4 (8)	3 (3)	0.2
Idiopathic	13 (26)	74 (76)	**<0.0001**
**Age (years)**	45,5 ± 22,3	35,6 ± 24,1	**0.02**
**Revisions, n (%)** Infection Bleeding Insufficient drainage	6 (12) 2 (4) 2 (4) 2 (4)	17 (18) 4 (4) 0 (0) 13 (13)	0.5 0.99 0.1 0.09

**Table 2:** Postoperative symptom improvement according to treatment modality.

Symptom	STS ratio of improvement n (%)	ETV ratio of improvement n (%)	*p value*
Headache	21/24 (87.5)	42/47 (89.4)	0.99
Nausea	9/9 (100)	9/11 (81.8)	0.5
Cognitive impairment	14/19 (73.7)	37/48 (77.1)	0.8
Seizures	3/4 (75.0)	3/5 (60.0)	0.99
Gait disorder	13/20 (65.0)	48/50 (96.0)	0.2
Urinary incontinence	2/5 (40.0)	20/25 (80.0)	0.1
Visual acuity decrease	20/26 (76.9)	20/28 (71.4)	0.8
Papilledema	17/25 (68.0)	21/26 (80.8)	0.3

### 3.1 Evaluation of the ETVSS in the STS group

**Fig. 3:**
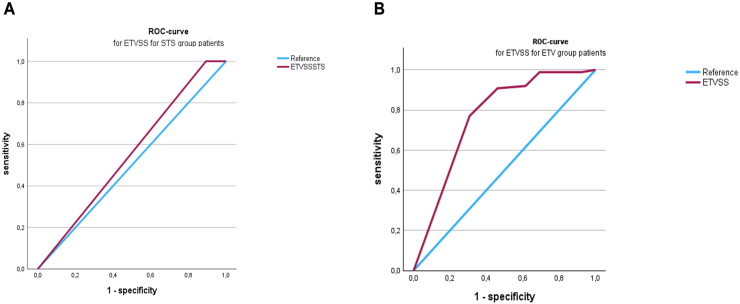
A: ROC analysis for the STS group. B: ROC analysis for the ETV group.

50 patients with a mean age of 45.5 ± 22.3 years underwent STS. The gender distribution was balanced, with 52% male patients. 33/50 (66.0%) patients had a solid tumor and 4/50 (8.0%) patients had a cystic tumor that led to aqueductal stenosis, while 13/50 (26%) patients had an aqueductal arachnoidal web causing the aqueductal stenosis. There were no patients with previous shunts, infections or bleedings in the STS group. Only 4/50 patients (8%) were below the age of 10 years. The mean follow-up period was 29.4 ± 27.4 months, with 2/50 patients (4.0%) undergoing revision surgery due to inadequate hydrocephalus treatment. The mean ETVSS was 88.2 ± 3.9% for the whole STS cohort. The 2 patients undergoing revision surgery had a mean ETVSS of 81.7 ± 4.1% compared with 89.1 ± 2.9% in successfully treated patients (p = 0.23). In univariate analysis, younger patient age (p = 0.05) and male sex (p = 0.04), were associated with a lower ETVSS. Age was confirmed as risk factor for treatment failure in the multivariate analysis (Table 3). ROC analysis revealed an AUC of 0.553 (Fig. 3A). Therefore, the ETVSS could not reliably predict treatment success in our cohort of patients undergoing STS.

**Table 3:** Uni- and multivariate analysis for risk factors influencing the ETVSS for patients treated with STS.

variable	odds ratio	95% CI	*p value*
Univariate analysis			
Evans index	0.1	−0.041 - 0.9	0.8
Diameter “clivus-basilar artery”	1.8	−13.4 - 7.1	0.2
Age	4.0	−244.8 - 74.5	0.05
Sex (m vs. f)	4.1	−5.9 - 81.5	0.04
Pre-OP symptoms			
Headache	0.2	−2.0 - 4.6	0.6
Nausea	0.1	−1.9 - 3.2	0.7
Cognitive impairment	0.1	−3.3 - 3.1	0.8
Seizures	3.1	−0.1 - 3.4	0.08
Gait disorder	1.1	−1.1 - 5.3	0.3
Visual abnormalities	0.05	−2.4 - 4.2	0.8
Urinary incontinence	1.2	−2.9 - 3.0	0.3
Multivariate analysis			
Age	2.1	0.001 - 3.1	0.04
Sex (m vs. f)	1.0	−1.0 - 3.0	0.3

### 3.2 Comparison of the ETV cohort with the STS cohort

97 patients underwent ETV. Tumors as the underlying pathology for aqueductal stenosis occurred more frequently in the STS group (STS 22.4% vs. ETV 13.6%, p < 0.0001). In addition, there were significantly more infants and newborns in the ETV group, resulting in a significantly lower mean age of 35.6 ± 24.1 years in the ETV group (p = 0.02). Apart from these differences, the demographic data of both cohorts were comparable. In the ETV group, there were two neonates with previous infections, but no patients with previous shunts or bleeding. The mean ETVSS in the ETV cohort was 81.8 ± 16.7% and was thus significantly lower than in the STS cohort (p < 0.01). In the ETV cohort, there were 13/97 revision surgeries (13.4%) due to inadequate hydrocephalus drainage. In the univariate analysis, younger patient age (p = 0.04) and a reduced distance between the basilar artery and clivus (p = 0.02) were associated with a lower ETVSS, while younger patient age was significantly striking in the multivariate analysis (Table 4). The mean ETVSS for successful ETV was 84.1 ± 13.5% compared to 71.7 ± 24.8% for failed ETV (p = 0.1). The ROC analysis yielded an AUC value of 0.766 for the ETVSS in the ETV group (Fig. 3B).

**Table 4:** Uni- and multivariate analysis for risk factors influencing the ETVSS for patients treated with ETV.

variable	odds ratio	95% CI	*p value*
Univariate analysis			
Evans index	0.06	0.2 - 0.7	0.9
Diameter “clivus-basilar artery”	5.9	−0.2 - 6.11	0.2
Age	4.3	−53.4 - 44.4	**0.04**
Sex (m vs. f)	0.4	−0.2 - 2.65	0.5
Pre-OP symptoms			
Headache	0.04	0.02 - 1.0	0.8
Nausea	0.6	−0.09 - 0.6	0.4
Cognitive impairment	0.002	−0.002 - 1.0	0.9
Seizures	0.06	−0.1 - 0.3	0.8
Gait disorder	0.7	−0.2 - 0.8	0.4
Visual abnormalities	2.2	0.2 - 3.2	0.1
Urinary incontinence	1.2	−0.1 - 1.4	0.7
Multivariate analysis			
Age	1.1	−0.8 - 2.7	0.3
Sex (m vs. f)	3.0	0.06 - 3.2	0.06 - 3.2

### 3.3 Exploration of new scoring systems

Despite using different statistical approaches, it was not possible to develop an alternative scoring system especially for the group of STS patient. Since only young patient age could be identified as risk factor for treatment failure, different models failed to predict treatment success.

## 4 Discussion

The ETVSS has been developed to predict the probability of successful treatment in patients with ETV for aqueductal stenosis (Kulkarni et al., 2009, 2010). The aim of this study was to evaluate the predictive value of ETVSS for STS in patients with aqueductal stenosis in order to facilitate the decision regarding the treatment modality.

We found that the ETVSS was unable to predict successful ETV in our STS cohort. In the ETV cohort, ROC analysis showed better sensitivity and specificity with an AUC value of 0.766, which is comparable to previous studies describing moderate predictive power of ETVSS. Ferreira Furtado et al. (2021) report an AUC value of 0.668 in a large cohort of pediatric patients. Given that age has a major influence on ETVSS and the number of children under 10 years of age in the STS cohort was very low, the negative results could also be due to the characteristics of the cohort. Although frame-based stereotactic surgery has been shown to be safe in children (Furlanetti et al., 2015; Schmutzer-Sondergeld et al., 2024b; Kirchleitner et al., 2025), endoscopic surgery is often preferred to avoid the stereotactic frame. In addition, there were no previous shunts or hydrocephalus due to infection or hemorrhage in the STS cohort, and the number of failed STS was very low at only 4%, which could also have an impact on the statistics. To confirm that the ETVSS is not suitable for patient selection, the score needs to be evaluated in a more heterogeneous cohort.

Uni- and multivariate analyses were performed to determine possible influencing factors associated with reduced ETVSS in the respective cohorts. In the ETV group, we found a small clivus to basilar artery diameter to be associated with treatment failure. This could reflect the surgical challenge of safely creating an adequate stoma in a very small and delicate area. However, even with an adequate stoma, the anatomy of a small prepontine cistern itself may influence CSF dynamics. Preoperative imaging should be studied in detail in this regard. Additionaly, younger patient age was associated with a lower ETVSS. In the STS cohort, also patient age was associated with a lower ETVSS. These results are consistent with the strong influence that age has on the calculation of the ETVSS and appear to be relevant for both ETV and STS. This may reflect the complexity of hydrocephalus in young children and also suggest that catheter placement does not necessarily overcome the suspected underlying problems of new arachnoid membrane formation, new ependymal or gliotic tissue formation in these young patients (Mohanty et al., 2002).

When considering other risk factors for stoma failure in ETV patients, these may also apply to STS patients. Posthemorrhagic hydrocephalus carries the risk of malabsorption and occlusion of catheter perforations after STS, and postinfectious hydrocephalus may be associated with a higher risk of delayed infection due to the use of foreign material. In contrast, STS can be a good alternative for non-tectal brain tumors, allowing stereotactic biopsy and treatment of hydrocephalus to be performed in a single stereotactic procedure, as Niedermeyer et al. have already reported in a cohort of 38 patients (Niedermeyer et al., 2023). This procedure led to a low ventriculoperitoneal shunt dependency during follow-up period and carried a remarkably low rate of surgical morbidity of only 2.6%.

The patient population in our study reflects the broad spectrum of patients who have TVH due to AS, with 40% of patients suffering from tumor-associated and 60% from idiopathic AS. We state that more patients with tumor-associated AS were treated by STS. This was most likely due to the fact that patients primarily presented to neuro-oncology specialists who had more experience with stereotactic rather than endoscopic techniques and were therefore more in favor of STS. On the other hand, patients with idiopathic TVH presented more frequently to the hydrocephalus specialists in our neurosurgical department who had more experience with ETV leading to a selection bias. However, subgroup analysis demonstrated that demographic data, the surgical complications as well as the symptomatic outcome did not differ depending on the etiology of AS.

Imaging-based therapeutic success, defined as the appearance of a new flow void, can only be documented in the ETV group, whereas this is not possible in the STS group. Similarly, this is not possible in studies comparing ETV with the conventional VP shunt in TVH (Cinalli et al., 2011; Konar et al., 2024; Kulkarni et al., 2017; Spennato et al., 2013). We have already explained this in our previous study (Ueberschaer et al., 2025)*.* However, the option of a stereotactic shunt into the prepontine cistern represents an alternative to ETV or the classic VP shunt, in which a flow void cannot and does not need be documented by imaging morphology, since treatment failure can be objectively determined by new or persistent symptoms or a lack of ventricular volume reduction.

Long-standing overt ventriculomegaly in adults (LOVA) is a differential diagnosis for TVH in cases of aqueductal stenosis. This is a chronic form of hydrocephalus that likely develops in childhood but does not become symptomatic until adulthood. Characteristic features include massive ventricular enlargement, obliterated cortical sulci, an enlarged or destroyed sella turcica, as well as optional stenosis in the aqueductal region on imaging, macrocephaly, and symptoms such as headaches, gait disturbances, dementia, parkinsonism, or incontinence. It is assumed that the condition has existed since childhood but, due to neural plasticity, is compensated for over a long period before decompensating in adulthood (often between the ages of 20 and 70) and leading to clinical symptoms (Mani et al., 2025; Montemurro, 2024; Montemurro et al., 2022; Palandri et al., 2021). Treatment is usually by surgery, often involving ventriculoperitoneal shunts (VPS) or ETV. VPS surgery is considered first-line therapy in the literature (Montemurro et al., 2022; Gillespie et al., 2022; Ved et al., 2017; Oi et al., 2000). However, the risk of overdrainage in LOVA patients must always be noted here, especially when fixed differential pressure valves (DPV) are used compared to programmable valves (PPV) (Kiefer et al., 2005)*.* The other treatment option for this condition is ETV, which has also shown good results (Kiefer et al., 2005). There are relatively few studies comparing ETV with shunt surgery in these patients. In general, both surgical procedures lead to comparable outcomes and symptom improvement; however, it should be noted that VPS is associated with a higher rate of infections or shunt dysfunction due to the implanted foreign material (Mani et al., 2025). No study has examined the implantation of a stereotactic internal shunt into the prepontine cistern in patients with LOVA so far, which could represent another interesting approach.

An important limitation of this study is the heterogeneity of the two treatment groups and the retrospective study design. For example, very few children under the age of 10 were included in the STS group, and this cohort was also significantly smaller than the ETV group. This results in poorer performance of the ETVSS in the STS group. Future studies should, on the one hand, be designed prospectively and, on the other hand, ensure a more homogeneous allocation to the two surgical techniques (ETV and STS) in order to achieve better comparability between the two techniques and ultimately to apply the ETVSS as well. Nevertheless, it must be emphasized that this study is the first of its kind to attempt such a comparison between ETV and STS, even if the results are negative.

Despite extensive statistical efforts, we were unable to find an alternative scoring system that would be suitable for STS patients or for both STS and ETV patients. This is most likely due to the small number of unsuccessful operations and the homogeneity of the cohort, in which only the young age of the patients was a preoperative risk factor for treatment failure. Future studies must aim to identify additional risk factors for treatment failure in STS patients in order to develop and validate an alternative scoring system. For patient selection, we must therefore adhere to our previously published scheme (Ueberschaer et al., 2025) and take into account the individual preferences of the neurosurgeons.

## 5 Conclusion

The ETVSS represents a tool to evaluate the risk of stoma occlusion after ETV in patients with aqueductal stenosis. In our cohort, the ETVSS did not enable a precise prediction of successful treatment of hydrocephalus by STS. However, its prediction was good in patients undergoing ETV. In patients with low ETVSS, individual decision-making is essential to adapt therapy options and to ensure the best possible management of hydrocephalus in the event of treatment failure. The evaluation of the ETVSS in a more heterogeneous and larger cohort in prospective studies might lead to different and perhaps promising results and may provide an opportunity to develop a new scoring system for patients who are eligible for STS.

## Consent to participate

Informed consent was obtained from all individual participants included in the study.

## Code availability

Not applicable.

## Authors' contributions

All authors contributed to the study conception and design. Data collection and analysis were performed by M. U., K. W. and M. S.-S. The first draft of the manuscript was written by M. U. and M. S.-S. M. U. and M. S.-S. and all authors commented on the previous versions of the manuscript. All authors read and approved the final manuscript.

## Ethics approval

All procedures performed in studies involving human participants were in accordance with the ethical standards of the institutional and/or national research committee as well as with the 1964 Helsinki declaration and its later amendments or comparable ethical standards. This study was approved of the institutional review board of the Ludwig-Maximilians-University Munich (reference number 22-0511).

## Consent for publication

Informed consent was obtained from all individual participants included in the study.

## Funding

The authors did not receive support from any organization for the submitted work.

## Conflicts of interest

All authors declare that they have no conflict of interest. There are no financial and personal relationships with other people or organizations that could inappropriately influence (bias) their work. The manuscript entitled “ Evaluation of the Endoscopic Third Ventriculostomy Success Score for Stereotactic Prepontine Stenting in Patients with Aqueductal Stenosis” has not been previously published in whole or in part or submitted elsewhere for review.

## Data Availability

The datasets used and/or analysed during the current study are available from the corresponding author on reasonable request.
